# An Investigation of Silica Aerogel to Reduce Acoustic Crosstalk in CMUT Arrays

**DOI:** 10.3390/s21041459

**Published:** 2021-02-19

**Authors:** Varshitha Yashvanth, Sazzadur Chowdhury

**Affiliations:** Electrical and Computer Engineering Department, University of Windsor, Windsor, ON N9B 3P4, Canada; yashvan@uwindsor.ca

**Keywords:** CMUT array, acoustic crosstalk, Scholte wave, crosslinked silica aerogel, biomedical, NDE

## Abstract

This paper presents a novel technique to reduce acoustic crosstalk in capacitive micromachined ultrasonic transducer (CMUT) arrays. The technique involves fabricating a thin layer of diisocyanate enhanced silica aerogel on the top surface of a CMUT array. The silica aerogel layer introduces a highly nanoporous permeable layer to reduce the intensity of the Scholte wave at the CMUT-fluid interface. 3D finite element analysis (FEA) simulation in COMSOL shows that the developed technique can provide a 31.5% improvement in crosstalk reduction for the first neighboring element in a 7.5 MHz CMUT array. The average improvement of crosstalk level over the −6 dB fractional bandwidth was 22.1%, which is approximately 5 dB lower than that without an aerogel layer. The results are in excellent agreement with published experimental results to validate the efficacy of the new technique.

## 1. Introduction

The capacitive micromachined ultrasonic transducer (CMUT) was invented in the early 1990s. They operate on electrostatic principles and offer attractive functional characteristics, such as higher coupling coefficient, higher fractional bandwidth, lower mechanical impedance, lower internal loss, better thermal dissipation, ease of batch fabrication, and easier integration with microelectronic integrated circuits [1,2,3,4]. However, even approximately after 30 years, CMUTs are not yet adopted as the main transducer type for ultrasound applications (e.g., imaging, therapy, nondestructive evaluation (NDE)) in the industry due to some serious inherent problems, such as crosstalk, dielectric charging, and center frequency drift [5,6,7,8].

The acoustic crosstalk in CMUT arrays occurs because of acoustical energy interaction among the elements and constituent CMUT cells in a CMUT array during transmission and reception. This unwanted coupling of acoustical energy modifies the deflection characteristics of effected CMUT cell diaphragms and has a significant impact on the overall array performance that includes the following: frequency response, acoustic radiation power, fractional bandwidth, signal-to-noise ratio, and beamforming [7,8,9,10,11,12].

The known physical phenomena that contribute to the acoustic crosstalk among the CMUT elements in an array are the dispersive guided modes comprised Scholte wave, leaky Rayleigh wave, A0 and S0 Lamb waves, and antisymmetric membrane mode propagating at the CMUT-fluid interface [5,7,9,10,11,12,13,14,15].

The author in [9] cited experimental results to conclude that the crosstalk results in poor angular response and range resolution due to an artificial increase in the effective element aperture of a CMUT array that compromises the axial resolution and introduces near-field artifacts to degrade image quality. In [10], it has been mentioned that “Crosstalk effects can cause the center cell or element in an array to become artificially weighted higher than the other elements in the array during the operation or even cause non-actuated elements to emit or receive ultrasound to result in inaccuracies during imaging, therapeutics, and NDE applications.”

It is worth mentioning here that the kerf width plays a crucial role in the intensity of crosstalk. As the kerf width in a low-frequency array is large due to a large wavelength, the intensity of crosstalk is relatively lower due to a longer travel distance for the interface waves. High resolution imaging needs high-frequency arrays where the kerf width is smaller due to a smaller wavelength. Consequently, the crosstalk level is higher. The authors in [8] identified crosstalk as a serious issue for emerging specialized imaging modes, such as high frequency high resolution photoacoustic imaging intended for noninvasive high-resolution deep-tissue imaging [8].

Researchers are pursuing different approaches to reduce acoustic crosstalk in CMUT arrays. In [5], it has been proposed to operate the CMUTs in pull-in mode to reduce the crosstalk effects. In another approach [7], acoustic bandgaps were introduced by fabricating nonfunctional passive cavities between the adjacent CMUT elements in an array. The membranes in those passive cavities have a cut-off frequency at or near the crosstalk frequency to prevent or mitigate the propagation of the Scholte waves [7]. The authors in [7] claimed that “this approach resulted in crosstalk reduction of approximately 10 dB in conventional operation mode (not the pull-in mode) without any damaging effect on generated acoustic pressure.” However, this technique adds additional fabrication complexity that affects size and cost. Moreover, this technique presents a challenge to fabricate high frequency CMUT arrays where the kerf width is very small.

Depositing a polydimethylsiloxane (PDMS) or parylene thin layer on the top of a CMUT membrane has been investigated in [5] and [9] to reduce unwanted coupling of acoustical energy in CMUT arrays. However, the presented experimental results in [5] and [9] reveal no significant improvement. The authors in [5] concluded that “The coverage of the PDMS reduced the phase velocity for the conventional and the collapsed operations, but did not significantly affect the crosstalk amplitude.” On the other hand, the PDMS layer used in [9] was much thicker (150 µm) compared to the one used in [5] to encapsulate the CMUTs to prevent propagation of the acoustic interface waves across the CMUT elements. This technique reduced the crosstalk levels by approximately 4 dB. However, the thickness of the PDMS layer is significantly high compared to the typical thickness of a CMUT diaphragm (0.2–3 µm).

In [14], it has been shown that introduction of a “double periodicity” in the layout of cells in a CMUT array can minimize acoustic crosstalk to some extent. To introduce the double periodicity, the authors added an additional kerf width between two elements to increase the distance between the elements. A numerical study presented in [15] showed that if the transmit waveforms of the adjacent CMUT elements can be altered, it is possible to mitigate acoustic energy coupling in CMUT arrays. However, dynamic programming of the waveforms depends on the application type, bandwidth of operation, and the capability of the signal processing hardware and warrant further exploration of this technique.

A 2.7 MHz 62 × 62 2-D array was theoretically investigated for crosstalk reduction using a row-column address method in [16]. The authors reported −23.9 ± 3.7 dB crosstalk for the nearest neighbor during linear operation and −40.2 ± 3.5 dB crosstalk when the array was used as columns-transmitting-rows-receiving mode. As the frequency is relatively low, the element spacing is higher; thus, the crosstalk level is lower compared to arrays operating at a higher frequency. The investigated row-column addressed method has been proposed for volumetric imaging using CMUT arrays. Experimental measurements from another row-column addressed array operating in 3 MHz center frequency reported −28.4 ± 2.4 dB average crosstalk at the nearest neighbor [17].

In [18], a boundary element based study has been presented to evaluate the effect of crosstalk in a 40 MHz 1-D CMUT array due to fluid coupling. The study concluded that crosstalk induces higher order vibrational modes in CMUTs. The higher-order vibrational modes contribute to additional resonant peaks and dips within the operating bandwidth. The authors further examined the pressure noise spectrum during the receive operation of a CMUT array and reported that crosstalk degraded signal-to-noise ratio (SNR) during the receive operation and contributed many local maxima in pressure noise spectrum.

The authors in [19] included crosstalk in the analysis of a large signal model of a 9 MHz CMUT array that they have developed; however, no quantified value of crosstalk was presented.

To summarize, all of the mentioned approaches involve either modification of CMUT standard operation, or necessitate additional fabrication steps, or require modifications of array geometry, or introduce additional circuit complexity and signal processing. Since such modifications alter standard operation and geometry of CMUT arrays while introducing additional fabrication complexity and costs, there is a need to investigate other solutions to reduce the crosstalk effects in CMUT arrays without any compromise of array geometry, fabrication, and cost.

Experimental results published in [20] established that when Scholte waves at a fluid-solid interface in an acoustic borehole encounter permeable formations, “viscous dissipation causes attenuation of wave amplitude and an increase in wave slowness.” This experimental fact opens up the possibility of introducing a permeable layer at the CMUT-fluid interface to attenuate and slow down the Scholte waves to reduce coupling of acoustical energy among the cells and elements of a CMUT array.

This study investigates the efficacy of fabricating a thin highly porous nanostructural permeable layer such as silica aerogel [21,22,23,24] on the top of a CMUT array to reduce the intensity of the Scholte waves at the CMUT-fluid interface using COMSOL™ multiphysics simulation software (Stockholm, Sweden) [25] based 3D finite element analysis (FEA) simulations. The simulation results were compared with experimental results published in [5]. The comparison establishes the effectiveness of silica aerogel in reducing crosstalk in CMUT arrays that proves the hypothesis.

The following sections constitute the rest of the paper. Section 2 provides the scientific principle behind the proposed approach, description, and set up of the CMUT array model for COMSOL 3D FEA simulation, 3D FEA simulation results with and without an aerogel coating, Section 3 summarizes the experimental validation of the proposed approach of crosstalk reduction, and finally, concluding remarks and future directions are provided in Section 4.

## 2. Materials and Methods

### 2.1. Scientific Rationale for Using Silica Aerogel

**Figure 1:**
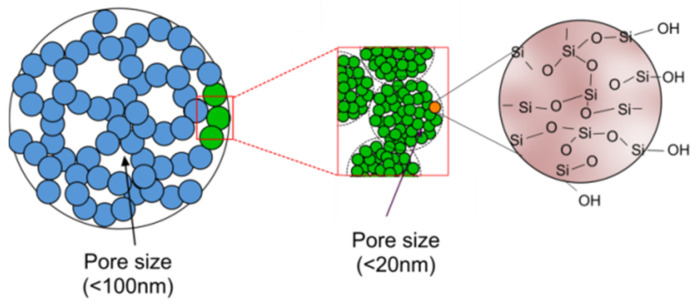
Structural network of silica aerogel [21].

The scientific principle behind the proposed approach lies on the fact that the microscopic structure of silica aerogel is characterized by high specific surface area of 500–1200 m^2^/g and a high porosity (80–99.8%) as shown in Figure 1 [21].

The mean pore diameter of silica aerogel has been reported as 20 nm and the primary particle diameter has been reported as 2–5 nm [22]. Such a nanoporous structure presents a highly complex zig-zag path with multiple reflection boundaries within a smaller geometry to a propagating acoustic wave. Consequently, an acoustic wave suffers multiple reflections within the aerogel network to rapidly loose energy within a short propagation distance. The acoustic propagation in such a nanoporous material depends on the interstitial gas nature and gas pressure [26] along with the density and texture of the structural network [27,28]. During propagation through a solid aerogel network filled with a gaseous substance, the wave is attenuated in both magnitude and speed as the wave energy is progressively exchanged between solid and gaseous media in a 3D space [29,30].

**Figure 2:**
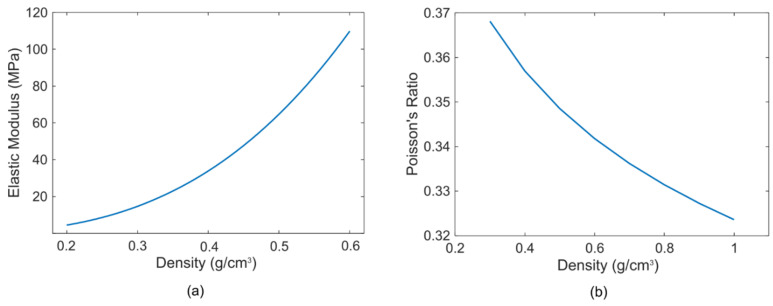
(**a**) Variation of elastic modulus with density, (**b**) Variation of Poisson’s ratio with density [21].

The physical properties of aerogel, such as Young’s modulus, Poisson’s ratio, ultimate strength in compression, and ultimate strength in tension depend on the fractal network of aerogel and aerogel density [31]. The Young’s modulus and Poisson’s ratio of aerogel is related to its density following:

(1) $$E\propto \rho^{3.11\pm 0.21},$$

(2) $$v=0.3236\cdot \rho^{-0.107},$$

where E is the Young’s modulus, v is the Poisson’s ratio, and ρ is the density. The relations are graphically shown in Figure 2 [21] as well. From Figure 2a, it can be seen that due to a lower density, aerogels exhibit a low elastic modulus and a low elastic modulus contributes to a low longitudinal speed following $c_{L}=\sqrt{E/v}$ [32]. Figure 2b shows the variation of Poisson’s ratio as a function of density that implies that a higher density contributes to a lower Poisson’s ratio to affect the plate modulus of an aerogel thin layer expressed as:

(3) $$\tilde{E}=\frac{E}{1-v^{2}},$$

which is generally about 10% larger than E [33]. Thus, both E and v contribute to the low sound speed in aerogel. The shear and rupture moduli scale with density accordingly.

These effects of density and Young’s modulus on the speed $c_{L}$ of a longitudinal sound wave in aerogel can be expressed following the scaling law in a compact manner as [34]:

(4) $$c_{L}\;\propto \;\rho^{1.3}.$$

**Figure 3:**
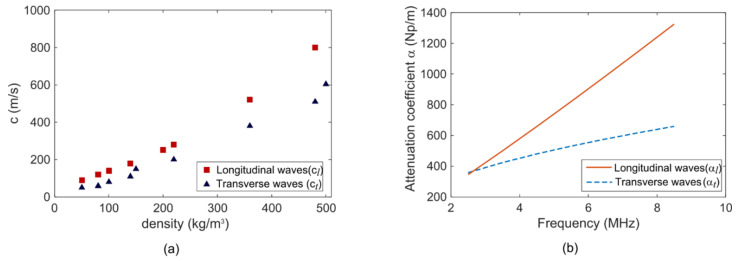
(**a**) Longitudinal ($c_{L}$) and transverse ($c_{T}$ ) sound speed in aerogel as a function of density. (**b**) Attenuation coefficients ($\alpha_{L}$ and $\alpha_{T}$ ) in the 5–10 MHz frequency range in aerogel [21].

As the density of aerogel is very low (0.05–0.4 g/cm^3^), the speed of a longitudinal sound wave propagating through an aerogel network is low as well, typically in the range of 100–500 m/s [22,24]. Figure 3a [21] shows both longitudinal and transverse speed of sound waves in aerogel as a function of density.

The attenuation coefficients of the longitudinal wave ($\alpha_{L}$) and transverse wave ($\alpha_{T}$) are functions of frequency [35] and can be expressed following:

(5) $$\alpha_{L}(f)=\alpha_{L1}(f),$$

(6) $$\alpha_{T}(f)=\alpha_{T1}f^{0.5\pm 0.15},$$

where $\alpha_{L1}=1.2\times 10^{-4}$ Np/m-Hz and $\alpha_{T1}=0.226\;\mathrm{Np}/m/\sqrt{\mathrm{Hz}}$.

The calculated longitudinal and transverse attenuation coefficients $\alpha_{L}$ and $\alpha_{T}$ for acoustic waves within an arbitrary frequencies range of 2.5–10 MHz are shown in Figure 3b [21]. The Figure 3b shows that both $\alpha_{L}$ and $\alpha_{T}$ increases with frequency; however, $\alpha_{T}$ is less sensitive to frequency variation compared to $\alpha_{L}$. Thus, a thin layer of silica aerogel at the CMUT-fluid interface will attenuate and slow down the Scholte wave (or any other interface wave) rapidly to contribute to a weak coupling among the CMUT elements.

The functionality of an aerogel coating can be enhanced further by frequency selective optimization of the longitudinal and transverse sound speed by controlling the pH level during the polymerization process [36]. As the size of the nanopores (volume fractal porosity) depend on the pH level of the precursor solution, the latter can be controlled to optimize the nanopore size to obtain a desired density and elasticity that in turn dictates the longitudinal sound speed in aerogel. The effect of the altered sound speed on the attenuation can be evaluated following Stokes’s law [37]:

(7) $$\alpha =\frac{2\eta \omega^{2}}{3\rho c^{3}}$$

where η is the dynamic viscosity coefficient of air, ω is the radian frequency, and c is the speed of sound.

However, in [36] it has been mentioned that “physics or chemistry relating percolation to kinetic growth over a wide pH range is absent.” Thus, a trial and error method can be followed to optimize the pore size for a particular frequency of interest. The nanopore size can be measured after fabrication using an X-ray scattering technique to determine a characteristic length and scattering angle [36].

Incorporation of an aerogel layer at the CMUT-fluid interface offers additional advantages. As silica aerogel is an excellent thermal insulator (0.01 W/m-K), a nominally thick silica aerogel layer can realize an excellent thermal insulator to prevent heating of the CMUT surface. Due to its extremely light weight, the added mass of a silica aerogel layer will have a negligible effect on the vibrational characteristics of a CMUT diaphragm. This feature is an excellent advantage, as in some other approaches, e.g., PDMS and parylene, the thickness of a protection or lossy layer on top of the CMUT diaphragm alters the diaphragm’s vibrational characteristics to affect resonant frequency and radiation pressure. Experimental investigations in [21,22,23,24] established that diisocyanate crosslinked silica aerogel films exhibit higher mechanical strength and hydrophobicity. In [23], it has been mentioned that the Google Nexus 7 body was made of a mechanically strong aerogel variant.

Thus, use of a silica aerogel layer can provide sufficient mechanical strength at a light weight; act as a good electrical and thermal insulator, and most importantly, can reduce crosstalk.

### 2.2. Simulation Model Description

**Figure 4:**
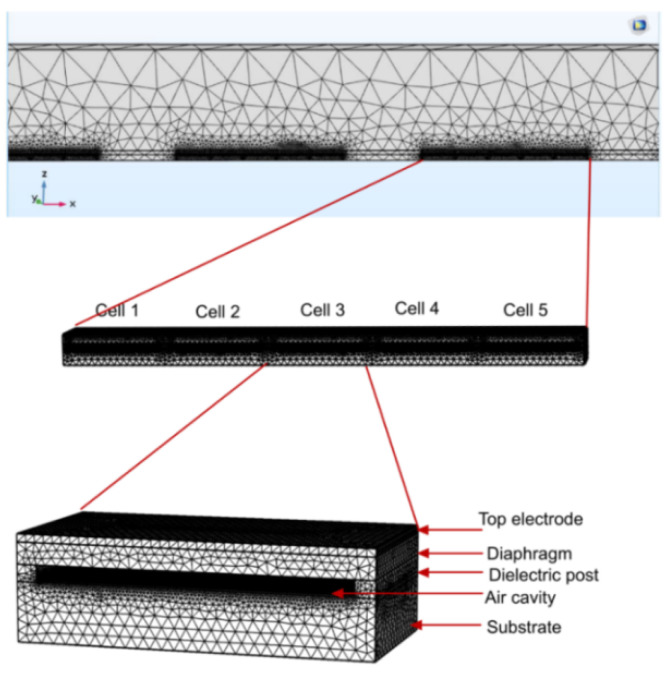
Simulation model of the capacitive micromachined ultrasonic transducer (CMUT) array in COMSOL™ [21].

To study the efficacy of silica aerogel in acoustic crosstalk reduction in CMUT arrays, 3D finite element model of a 5-element linear CMUT array was constructed in COMSOL. The FEA model is shown in Figure 4 [21]. Due to symmetry, only half of the array was modeled.

The maximum element size of meshes was set to be a one-sixth of the smallest wavelength in a frequency domain sweep. The diaphragm was set to be a linear elastic material and suitable boundary conditions were applied at the edges. The interface between the electromechanical and acoustic pressure domains was modeled as a fluid–solid coupled boundary, which corresponds to a pressure load on the electromechanical domain and a normal acceleration on acoustic pressure domain, respectively.

The array has a center frequency of 7.5 MHz and each element was constructed to have 5 CMUT cells. The array specifications and material properties are provided in Table 1 [21] and Table 2 [21].

### 2.3. Acoustic Crosstalk Levels without Silica Aerogel

**Figure 5:**
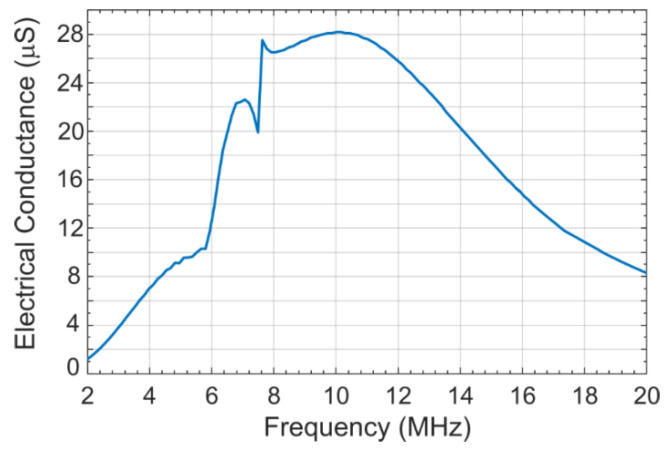
Frequency response of the excited CMUT array element without silica aerogel coating [21].

**Figure 6:**
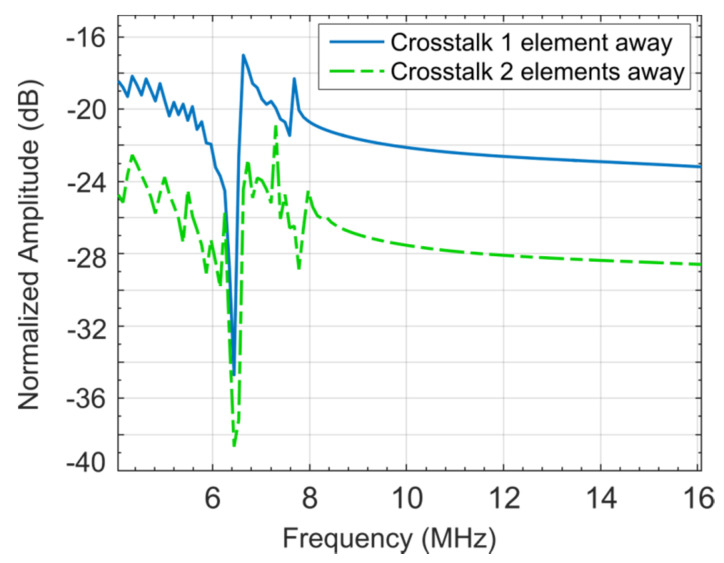
Crosstalk level at the nearest and the next neighbor without silica aerogel coating [21].

A coupled acoustic-structure analysis was conducted in COMSOL Multiphysics environment using the model described in the previous section. The COMSOL generated frequency response of the simulation model is shown in Figure 5 [21]. To calculate the crosstalk level in water, the model was excited with a 7.5 MHz center frequency signal with a bandwidth of −6 dB (4–16 MHz) using a 1 MHz frequency step. The peak and average crosstalk levels at the nearest-neighbor and next-nearest neighbor with respect to the excited element as calculated from normalized displacement and frequency response is shown in Figure 6 [21]. Figure 6 shows that for the first neighbor, the peak crosstalk level is −17.18 dB and the average crosstalk level over the −6 dB bandwidth is −22.44 dB. Corresponding standard deviation is 1.88 dB. For the second neighbor, the peak and average crosstalk levels are −21.07 and −27.46 dB, respectfully. The results are summarized in Table 3 [21].

As anticipated, the crosstalk levels decrease linearly as the elements move further away from the excited element. To validate the simulation study, the average crosstalk results are compared with experimentally measured crosstalk levels of a similar array published in [5]. The simulation results are found to be in excellent agreement with published experimental data to validate the accuracy of the executed FEA simulation.

### 2.4. Acoustic Crosstalk Levels with Silica Aerogel

To evaluate the effectiveness of aerogel in reducing crosstalk, a thin layer of silica aerogel has been added on the top of the simulation model. Out of different aerogel varieties, a silica aerogel specimen crosslinked with diisocyanate (known as x-Aerogel) has been chosen to ensure enhanced mechanical strength and durability for the target biomedical imaging and NDE applications.

**Figure 7:**
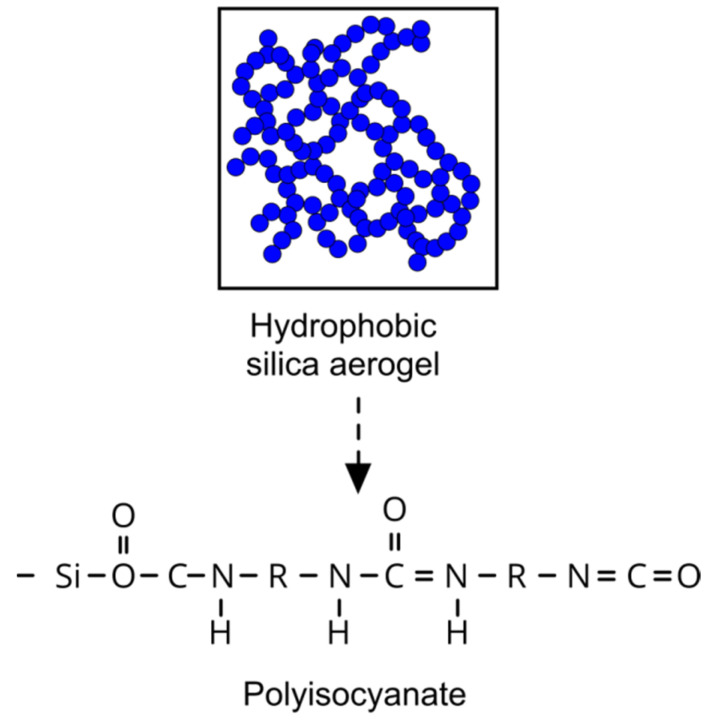
Reaction for amine modified aerogel crosslinking with diisocyanates [21].

A method presented in [19] was followed to obtain the desired mechanically strong structure of the cross-linked silica aerogel used in the simulation. The process starts with soaking a hydrophobic silica aerogel produced using an hourglass method in a solution containing diisocyanate crosslinking agents. The resulting solution is then heated slowly to evaporate dry and enable polymer linking as the diisocyanate agent starts to react and undergo bonding with the silanol group as shown in Figure 7 [21].

The chosen crosslinked silica aerogel layer can easily be fabricated cost-effectively on the top of the wafer after completing the metallization process using a common and inexpensive spin deposition method as described in [38,39,40]. The physical properties of the selected aerogel used in the simulation are provided in Table 4 [21].

#### 2.4.1. Aerogel Passivation Layer Thickness Determination

**Figure 8:**
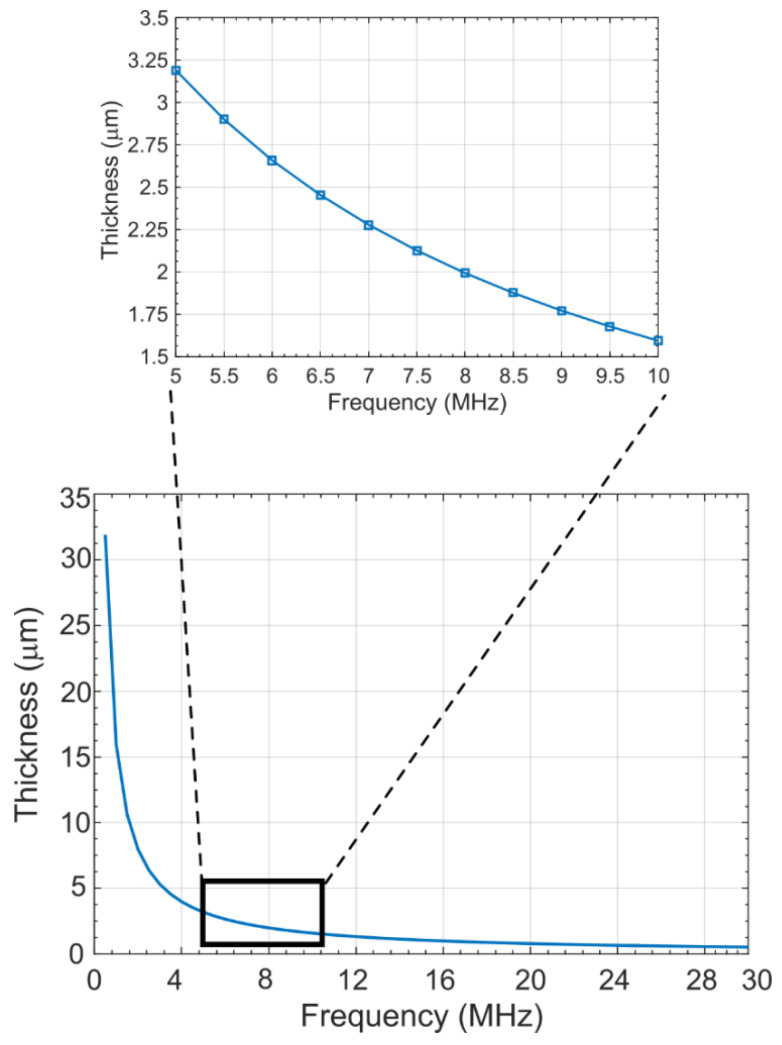
Coating thickness for various center frequency of operation [21].

A parametric optimization study was conducted to develop a method to determine the thickness of the aerogel layer as a function of center frequency to optimally suppress the acoustic crosstalk in a CMUT array. A sound speed of 1500 m/s in water was used in the analysis. Finally, a graph as shown in Figure 8 [21] was obtained that can be used to determine the thickness of the silica aerogel layer for any target center frequency of operation.

Figure 8 shows that the higher the center frequency, the lower the thickness of the aerogel layer necessary to optimally reduce the crosstalk. The reason for this lower thickness lies on the fact that the interface Scholte waves behave as non-dispersive waves below 12 MHz and travels without much reduction in wave energy. Thus, thicker passivation layers are necessary at frequencies below 12 MHz to ensure sufficient attenuation.

#### 2.4.2. Effect of Aerogel Layer on Diaphragm Deflection

**Figure 9:**
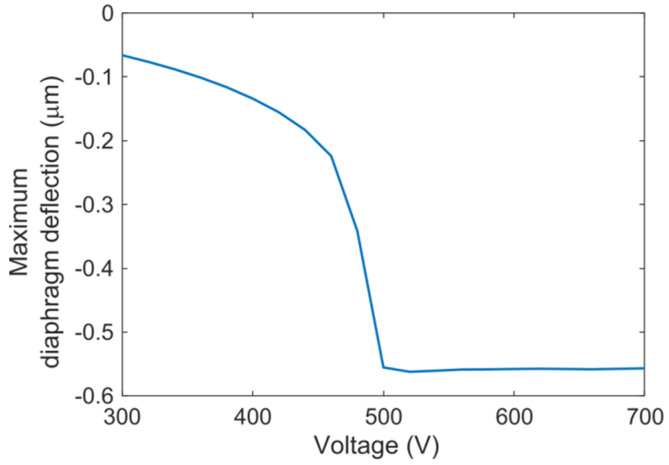
3D electromechanical finite element analysis (FEA) results showing the CMUT diaphragm deflection as a function of direct current (DC) bias [21].

To evaluate the effect of a silica aerogel layer of desired thickness on the load-deflection behavior of a CMUT cell, a 3D electromechanical FEA simulation was conducted using IntelliSuite™, a MEMS design tool from Intellisense™ [41]. Table 1 and Table 2 specifications were used for the simulated CMUT cell with the addition of a 2.1 μm thick silica aerogel layer. The thickness of the aerogel layer was determined from Figure 8 for a center frequency of 7.5 MHz. The FEA simulation results for the maximum deflection of the CMUT diaphragm as a function of the applied DC voltage are plotted in Figure 9 [21].

Electromechanical 3D FEA simulations were conducted to determine the pull-in (collapse) voltage of a CMUT cell in the model with and without any aerogel layer. In both the cases, the pull-in voltages were turned out to be 482 volts. These simulation results indicate that the aerogel coating has a negligible effect on the load-deflection behavior of the diaphragm.

#### 2.4.3. CMUT Arrays with Silica Aerogel Passivation Layer

**Figure 10:**
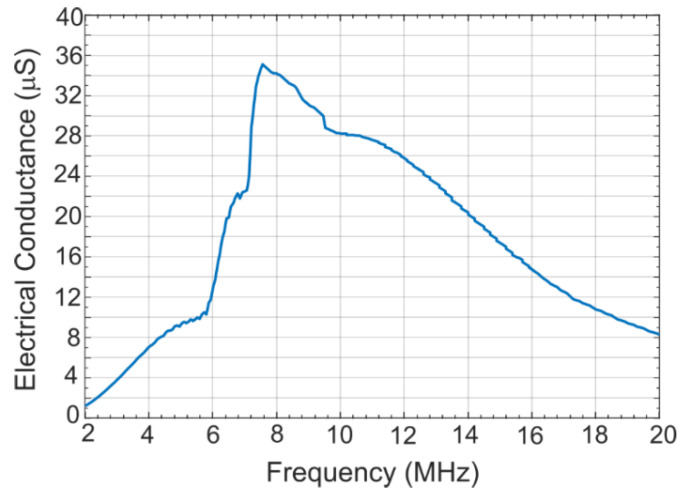
Frequency response of the excited element in an aerogel coated CMUT array [21].

**Figure 11:**
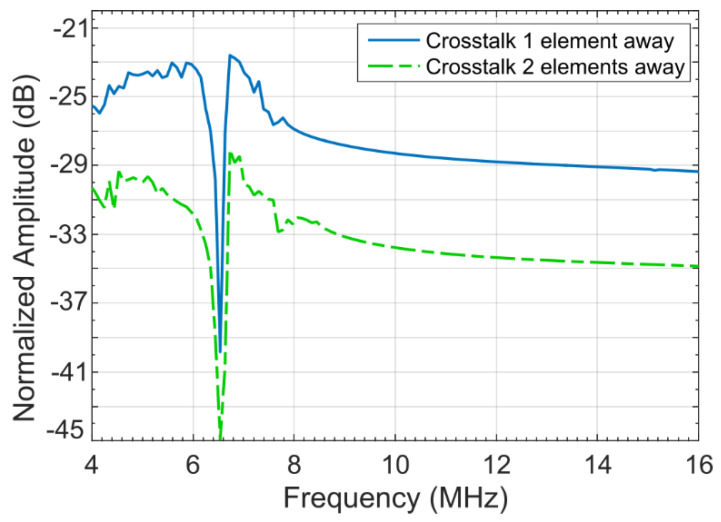
Crosstalk levels of an aerogel coated CMUT array [21].

Following Figure 8, for the 7.5 MHz simulation model in COMSOL, the thickness of the aerogel layer can be calculated as 2.1 µm. Figure 10 [21] shows the frequency response of the model in the COMSOL environment. The peak and average crosstalk levels at the nearest-neighbor and next-nearest neighbor with respect to the excited element were extracted from normalized displacements-frequency response shown in Figure 11 [21].

Following Figure 11, the peak crosstalk level for the first neighboring element is approximately −22.6 dB as compared to −17.18 dB for the model without an aerogel layer. The crosstalk level averaged over the −6 dB fractional bandwidth can be approximated from Figure 11 as −27.4 dB. This shows an improvement of 4.96 dB in crosstalk levels for the first neighboring CMUT element without an aerogel layer.

The peak and −6 dB averaged crosstalk levels for the next neighbor is −28.6 and −33.8 dB, respectively, as can be approximated from Figure 11. The improvement is 6.34 dB. Table 5 [21] summarizes the crosstalk levels for the first and the next neighboring elements for the CMUT model with an aerogel layer.

These results show the effectiveness of silica aerogel thin films in reducing acoustic crosstalk in CMUT arrays, validating the hypothesis and paving the way for other possibilities of crosstalk reduction as discussed later.

## 3. Results

### Experimental Validation of the Simulation Results of the Efficacy of Silica Aerogel in Crosstalk Reduction

Experimental validation of the COMSOL simulated crosstalk levels for the CMUT arrays with and without an aerogel layer was conducted by comparing the simulated results with experimental results published in [5]. The comparison is presented in Table 6 [21]. The comparison establishes that the proposed technique of coating the CMUT surface with a silica aerogel passivation layer of necessary thickness can reduce the peak crosstalk level for the first neighboring element in a 7.5 MHz array by approximately 5 dB that amounts to an improvement of 31.5% over a CMUT array operating in the same center frequency without an aerogel layer. The average improvement was 22.1% over the −6 dB fractional bandwidth.

Table 7 [21] shows a comparison of the −6 dB averaged crosstalk levels of aerogel coated CMUT arrays with the crosstalk levels of piezoelectric ultrasonic transducer arrays published elsewhere [42,43,44]. The comparison shows a significant improvement in crosstalk levels in CMUT arrays with the industry standard piezoelectric technology based ultrasonic transducer arrays.

Importantly, the reduction in crosstalk levels achieved following the proposed technique is without any modifications or compromise of the CMUT array geometry or fabrication process. The aerogel layer can easily be deposited and patterned on the top once the conventional CMUT fabrication is completed. Furthermore, the technique can be used for both conventional and collapse mode operation CMUT arrays.

The modification of a fabrication process previously developed by the authors [45] is in progress to include the necessary process steps to deposit and pattern the aerogel layer with the specifications listed above in Table 1, Table 2, Table 3 and Table 4. The experimental procedures developed and conducted by the authors [45,46] along with additional measurement steps will be followed to characterize the functionality of the complete array and the efficacy of the aerogel layer after fabrication. The fabrication and experimental results remain the subject of a future publication.

## 4. Conclusions

The investigation proves that a thin layer of nanoporous crosslinked silica aerogel at the CMUT-fluid interface can attenuate and slow down Scholte waves to reduce cross-coupling of acoustical energy among the cells and elements in a CMUT array to improve the quality of acoustic data acquisition. High quality data can provide superior NDE, therapy, and diagnosis. The technique can be used to realize high resolution high frequency CMUT arrays with reduced acoustic crosstalk for emerging new modes of ultrasound applications, e.g., photoacoustic imaging, as well.

Due to its extreme light weight, the effect of silica aerogel layer on the static and dynamic vibrational characteristics of a CMUT diaphragm is minimal to compromise any design objective and device performance while reducing fabrication complexity, and cost. Unlike other crosstalk minimization schemes, the proposed aerogel layer will also act as excellent thermal and electrical insulator. The developed technique can be optimized for any target Scholte wave frequency by altering the porosity of the aerogel layer through pH level control during polymerization.

The fundamental concept can be exploited further by creating nanopores or nanograting in a silicon dioxide or a polymer film to create a microfabricated artificial aerogel-like network with an optimized porosity factor to suppress acoustic interface waves further.

## Figures and Tables

**Table 1:** Specifications of the CMUT cells in the simulation model [21].

Parameter	Value	Unit
Cell sidelength, a	16	µm
Diaphragm thickness, $d_{m}$	1.3	µm
Gap thickness, $d_{0}$	650	nm
Insulating layer thickness, $d_{i}$	100	nm
Top electrode thickness, t	100	nm
Contact pad thickness, $t_{c}$	0.4	µm
Dielectric post thickness, $L_{c}$	2	µm

**Table 2:** CMUT array material physical properties [21].

Parameter	Benzocyclobutene (BCB) (Diaphragm, Dielectric Post)	Gold (Top Electrode)	Silicon (Substrate)<100>	Unit
Density, ρ	1050	19300	2329	kg/m^3^
Young’s modulus, E	2.9	70	165	GPa
Poisson’s ratio, v	0.34	0.44	0.26	
Residual stress, σ	28	106	55	MPa
Relative permittivity, ε	2.6	6.9	11.8	

**Table 3:** Crosstalk levels at the nearest and next-nearest neighbor in the simulated capacitive micromachined ultrasonic transducer (CMUT) array without aerogel coating [21].

Element Number	Peak Crosstalk Level (dB)	Average Crosstalk Level (dB)
1st neighbor	−17.18	−22.44
2nd neighbor	−21.07	−27.46

**Table 4:** Specifications of silica aerogel layer [21].

Parameter	Value	Unit
Density, ρ	0.4	g/cm^3^
Young’s Modulus, E	50	MPa
Poisson’s ratio, v	0.34	
Speed of sound, c	300	m/s

**Table 5:** Crosstalk levels of the aerogel coated CMUT array [21].

Element Number	Peak Crosstalk Level (dB)	Average Crosstalk Level (dB)
1st neighbor	–22.6	–27.4
2nd neighbor	−28.6	−33.8

**Table 6:** Experimental validation of the effectiveness of the aerogel layer in crosstalk reduction in CMUT arrays [21].

Parameter	Presented Design First Neighbor		Ref. [5]	
Type	CMUT without aerogel (simulated)	CMUT with aerogel (simulated)	CMUT without Polydimethylsiloxane (PDMS) (experimental)	CMUT with 5 µm PDMS (experimental)
Diaphragm material	BCB	BCB	Silicon Nitride	Silicon Nitride
Center frequency (MHz)	7.5	7.5	5.8	5.8
Number of elements	64	64	64	64
−6 dB Fractional bandwidth	133	114	130	100
Peak crosstalk level (dB)	−17.18	−22.6	−17	−17
Average crosstalk level (dB)	−22.44	−27.4	−23.2	−23.3

**Table 7:** Comparison of crosstalk levels of CMUTs with an aerogel coating and piezoelectric transducers [21].

Element Position	Average Crosstalk Level (dB)		
Transducer type	CMUT without aerogel layer (7.5 MHz)	CMUT with aerogel layer (7.5 MHz)	Lead zirconate titanate (PZT) transducers (1–10 MHz)
1st neighbor	–22.44	−27.4	−30
2nd neighbor	−27.46	−33.8	−38

## Data Availability

Data sharing not applicable.
